# Using machine learning methods and EEG to discriminate aircraft pilot cognitive workload during flight

**DOI:** 10.1038/s41598-023-29647-0

**Published:** 2023-02-13

**Authors:** Hamed Taheri Gorji, Nicholas Wilson, Jessica VanBree, Bradley Hoffmann, Thomas Petros, Kouhyar Tavakolian

**Affiliations:** 1 Biomedical Engineering Program, University of North Dakota, Grand Forks, ND USA; 2 Departments of Aviation, University of North Dakota, Grand Forks, ND USA; 3 Department of Psychology, University of North Dakota, Grand Forks, ND USA

**Keywords:** Biomedical engineering, Electroencephalography - EEG, Aerospace engineering, Human behaviour

## Abstract

Pilots of aircraft face varying degrees of cognitive workload even during normal flight operations. Periods of low cognitive workload may be followed by periods of high cognitive workload and vice versa. During such changing demands, there exists potential for increased error on behalf of the pilots due to periods of boredom or excessive cognitive task demand. To further understand cognitive workload in aviation, the present study involved collection of electroencephalogram (EEG) data from ten (10) collegiate aviation students in a live-flight environment in a single-engine aircraft. Each pilot possessed a Federal Aviation Administration (FAA) commercial pilot certificate and either FAA class I or class II medical certificate. Each pilot flew a standardized flight profile representing an average instrument flight training sequence. For data analysis, we used four main sub-bands of the recorded EEG signals: delta, theta, alpha, and beta. Power spectral density (PSD) and log energy entropy of each sub-band across 20 electrodes were computed and subjected to two feature selection algorithms (recursive feature elimination (RFE) and lasso cross-validation (LassoCV), and a stacking ensemble machine learning algorithm composed of support vector machine, random forest, and logistic regression. Also, hyperparameter optimization and tenfold cross-validation were used to improve the model performance, reliability, and generalization. The feature selection step resulted in 15 features that can be considered an indicator of pilots' cognitive workload states. Then these features were applied to the stacking ensemble algorithm, and the highest results were achieved using the selected features by the RFE algorithm with an accuracy of 91.67% (± 0.11), a precision of 93.89% (± 0.09), recall of 91.67% (± 0.11), F-score of 91.22% (± 0.12), and the mean ROC-AUC of 0.93 (± 0.06). The achieved results indicated that the combination of PSD and log energy entropy, along with well-designed machine learning algorithms, suggest the potential for the use of EEG to discriminate periods of the low, medium, and high workload to augment aircraft system design, including flight automation features to improve aviation safety.

## Introduction

While in flight, pilots may choose varying degrees of automation to support their operation, each degree having some impact on the cognitive workload of the pilot^1^. Additional contributions to cognitive workload and automation management include weather hazards such as poor visibility, thunderstorms, turbulence, and variable wind conditions^2,3^. During a routine flight, diverse environmental conditions, pilot experience, and variations in aircraft automation may induce cumulative variations in the workload experienced by the pilot(s). The combination of such factors can influence the cognitive workload and, subsequently, the pilot's ability to effectively fly the aircraft, manipulate the automation, and manage changes in the operational environment. Periodically, a pilot's cognitive workload may become higher than he or she is able to effectively manage, which can lead to increased reaction times, decreased performance, and increased risk for human error^4^. Conversely, a low cognitive workload has been shown to contribute to boredom and lack of attention^5,6^. Such cases are equally problematic for pilots and other stakeholders within private, military, or commercial aviation.

Pilot cognitive workload has been studied across many operational environments, including simulated flight with fighter pilots^7^, in simulated flight using dry-electrode EEG^8^, and in live flight in single-engine training aircraft^9^. Similarly, researchers found a connection between brain (EEG), eye motion (EOG), and heart rate variables during the transition between cognitive workload levels and eventually toward cognitive fatigue^10^. The cognitive workload can be quantified using various physiological methodologies or subjective measures^11^. Multiple factors contribute to cognitive workloads; some of the most obvious ones for aviators are the phase of the flight operation (e.g., “taxi” vs. “landing”), the environmental factors (turbulence, storms, or runway surface conditions), the complexity of the terminal environment (“New York JFK” versus “Omaha, Nebraska”), and the pilots' relative experience on the aircraft (from “novice” to “expert”). Although many factors may influence the pilots' cognitive workload, how these changes manifest into the effective operation of the aircraft and its automation remain an opportunity for continued investigation.

### Aircraft automation

Piloted aircraft may be designed and equipped with high degrees of automation, up to or including the ability to *autoland* at a given airport^12^. Additionally, all piloted aircraft also have the ability to be flown manually from engine-start to engine shut-down. In the case of primary training aircraft, such aircraft have almost no or very little designed automation, allowing student pilots consistent practice in managing the state of the aircraft through their own psychomotor control inputs. Given the wide variety of automation and pilot interfaces, it is important that we understand the fundamentals of pilot cognitive workload to better understand how these considerations may be designed into aircraft ranging from primary, single-engine training aircraft all the way to transport category passenger or cargo aircraft with multiple engines, high degrees of available automation and complicated systems. As we further refine these complex aircraft systems to improve aviation safety or operational efficiency, greater emphasis must be placed on the pilots' interactions with said systems. An effective system will focus on optimizing the human–machine interface, including changes in physiological markers, which may suggest optimum or less-than-optimum aircraft system design.

### Augmented cognition

Pilots operating aircraft during various workload conditions may benefit from a concept called “augmented cognition.” This concept can be simply described as a set of automation that allows the systems to augment or supplement aircraft systems, automation, or flight information display systems to improve flight safety.

Augmented cognition uses neurophysiological information supplied by the system operator, the pilot, in this case. One opportunity for further research includes continuous physiological data collection and processing from the pilot to assess attention, fatigue, task engagement, and stress^13^. From this information, changes in operator state can be used to induce changes in system design. In aviation, such changes could include active adaptations to the electronic flight information systems (EFIS) as well as induce changes in software logic or control systems of the autopilot, autothrust, or flight director systems. Broadly, such system design could have benefits across many domains, particularly relevant in safety-sensitive environments involving the rapid decision-making of humans and the machines they control.

Among several brain activity monitoring techniques, electroencephalography (EEG) has been shown to be one of the most popular techniques for monitoring cognitive workload because of its noninvasiveness and cost-effectiveness. In addition, its high temporal resolution allows the researcher to monitor and assess cognitive workload states in real-time^14,15^. More specifically, EEG measures the summation of postsynaptic action potentials, allowing for the interpretation and quantification of cortical activity in various brain regions^16^. Each EEG recording generates sinusoidal waves that, dependent upon frequency, are usually decomposed into five main sub-bands, namely Delta (0.5–4 Hz), Theta (4–8 Hz), Alpha (8–12 Hz), Beta (12–35 Hz), and Gamma (35–100 Hz)^17^.

Two prominent EEG frequency waves, alpha, and theta, have emerged as primary metrics of human performance. Alpha spectral band power is EEG oscillatory activity that fluctuates between 8 and 13 Hz, occurring over the entire scalp but most prominently in the parietal-occipital areas. Alpha reflects general cortical arousal in that low alpha is associated with a state of alertness, and high alpha is associated with relaxation or drowsiness^18^. Alpha has been shown to decrease with increased task difficulty^19^, as well as with increased memory load^20–22^. Frontal theta power increases with increased workload^23^ and increases with mental effort associated with a vigilance decrement^24^. In addition, increased theta power at anterior frontal or frontal midline areas, along with decreased alpha at parietal areas, has been associated with increased workload^23^.

Furthermore, the other more useful information that can be extracted from EEG signals is the ratio of different EEG sub-bands. In other words, the combination of such important information can be considered a potential indicator of cognitive workload^25,26^. For instance, frontal theta divided by parietal and occipital alpha (theta/alpha) has been proven to be an essential physiological indicator of cognitive workload and mental fatigue^27^. More specifically, an increase in cognitive workload has been linked to an increase in frontal theta power and a reduction in parietal alpha power^23,28^. Theta divided by beta (theta/beta) is another ratio considered an important index for assessing attention or cognitive processing^29^. Theta/beta is mainly studied in patients with attention deficit hyperactivity disorder (ADHD), in which an increasing ratio is associated with decreased cognitive performance^30,31^. Another index that can provide useful information for monitoring the cognitive workload level is diving beta by alpha plus theta (beta/alpha + theta), also named engagement index (EI). The studies have shown a positive correlation between beta activity and alertness and a negative correlation between alpha and theta activity and alertness which can be used to detect vigilance and mental fatigue^32,33^. Furthermore, this ratio could represent the information gathering, visual scanning, and sustained attention of subjects^13^.

With respect to the explanation mentioned above regarding the importance of assessing cognitive workload using EEG signals, monitoring the operator's functional state can determine when the operator is faltering and allow the introduction of adaptive aiding by implementing some form of automation. Attempts to implement adaptive aiding have utilized physiological triggering of adaptive aiding^34^.

One of the challenges of accurately monitoring the operator's state is to rapidly classify the operator's state of workload or diminished attention. A variety of machine learning techniques have been applied to this problem to discriminate participants' workload states into high or low accurately.

Blanco et al.^35^ provided evidence to support the utility of EEG-based measures of workload for distinguishing between flight activities of three levels of difficulty in simulated flight. The authors tested 21 United States Naval Academy students that were 18 to 23 years of age. All participants had previously experienced basic flight training and were tested under three different flight scenarios of differential levels of difficulty (easy, medium difficulty, and difficult). Each participant flew each scenario once for 10 min in counterbalanced order. Scalp EEG signals were recorded from Fz, FCz, Cz, and Pz using the International 10–20 system. The authors reported a strong negative correlation between behavioral performance and EEG workload measures. The mentioned study demonstrated that EEG measures can differentiate various workload levels during flight and that the workload measures were related to cognitive performance. However, a subset of subjects demonstrated increased cognitive workload without any decrement inflight performance. Several machine learning techniques were evaluated, and linear discriminant analysis (LDA) was the best-performing classifier.

In another research conducted by^25^, several EEG power sub-bands were examined to establish a set of indicators to evaluate the subjects' cognitive workload during the teleoperation of an unmanned aerial vehicle (UAV) (a simulation environment). Sixteen participants were recruited, and their subjective indicators of the perceived workload using the Air Traffic Workload Input Technique (ATWIT), heart rate (HR), and EEG were recorded. The joint mutual information (JMI) algorithm and LDA were employed as feature selection and classification techniques, respectively, and results showed that nineteen EEG features could lead to higher accuracy (82.23%) than ATWIT and HR for discrimination between different workload setups.

Although the results of the two studies mentioned above were compelling, they need to be extended from a flight simulator to any inflight situation. Therefore, the current study collected inflight physiological data from pilots while executing flight patterns that cause different workload levels. Some of the maneuvers, such as executing a missed approach at minimums and consecutive steep turns, can result in a higher workload. Whereas turn east/west-bound and top of the climb were classified as medium workload. Furthermore, taxiing at an un-towered airport and flying straight-and-level were categorized as low workload. So, the primary goal of this study was to demonstrate the applicability and reliability of EEG measures and the functionality of machine learning algorithms to determine the most suitable EEG indicator for distinguishing three classes of inflight pilot cognitive workload, low, medium, and high.

## Material and method

### Subjects

Ten individuals participated in this study, all of whom were undergraduate aviation students attending the University of North Dakota. The eligibility criteria required that all participants hold an FAA Class I or Class II medical certificate as well as a Federal Aviation Administration (FAA) commercial pilot certificate. All subjects were current in their respective aircraft type, whether a Piper Archer or Cessna 172S, and were familiar with the Garmin G1000 avionics system utilized in this experimental design. Each subjected self-reported their flight hours prior to participating in the study, the mean being 323.6, ranging from 170 to 840. All subjects were compensated for their participation, and data were collected in accordance with approved Institutional Review Board (IRB) procedures at the University of North Dakota. All methods were performed in accordance with the relevant guidelines and regulations.

### Experimental procedure

Before any study-related procedure at the airport, all participants were briefed on the study tasks and procedures and provided their informed consent to the study team. On the subsequent day of the study visit, all participants were tasked to complete questionnaires related to their demographics, education, recent sleep, and stimulant intake (i.e. caffeine) prior to the setup and application of the ABM EEG data collection system. The participant then completed three baseline activity readings per the ABM software guidelines. Following the baseline data collection, participants boarded the Piper or Cessna single-engine, four-seat training aircraft, all equipped with the common Garmin G1000 avionics system. The participant completed the pre-determined flight sequence at the study team's discretion, which comprised a safety pilot in the front seat and a research assistant in the back seat. A detailed item-by-item flight profile and data collection form may be found as an Supplementary material to this document. It is worth mentioning that the experimental protocol was standardized for each pilot participant and included as an appendix to this manuscript. This maneuver profile represents a varying degree of workload for the pilot participant based on the experience of the Federal Aviation Administration (FAA) certified flight instructor and PI of this study. For example, "straight and level flight" (maneuver items #8, #23) requires low pilot workload from the pilot, whereas intercepting and flying a precision approach (maneuver items 16/17 and items 30/31) require a high degree of attention and focus from the pilot. The workload required of these maneuvers is natural and obvious element of piloting an aircraft and is not dissimilar to the workload differences a driver of a car experiences while on an open highway versus navigating precisely through 'rush hour'.

**Figure 1:**
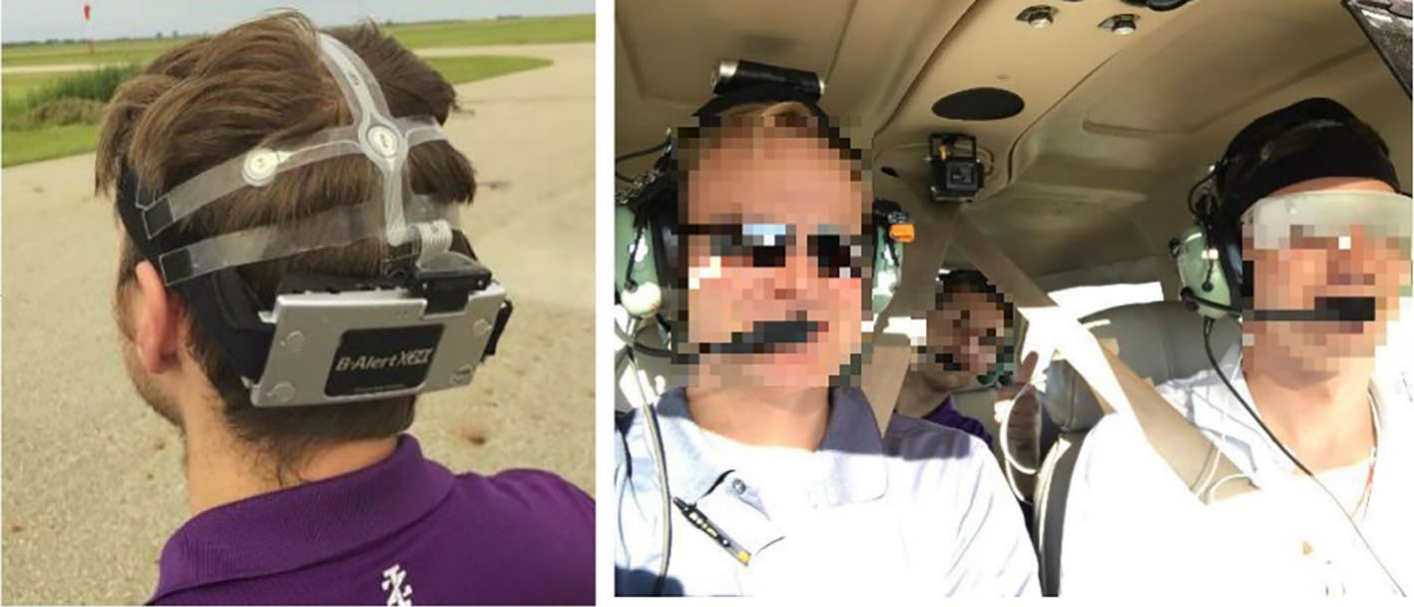
(Left) ABM B-Alert X24 system, (Right) Data collection during live flight (image previously referenced in^9^).

During the flight, the safety pilot and research assistant in the aft seat simultaneously recorded times of the tasks as the maneuvers progressed on the standardized maneuver list (Fig. 1 (right)). Additionally, each second (1 Hz), the Garmin G1000 avionics system records several dozen flight parameters onto an SD card. The recorded data from the G1000 system includes such items as altitude, heading, and airspeed engine rotations per minute (RPM) which were extracted from the aircraft and used along with the completed maneuver profile time sheets to derive accurate maneuver time periods for data analysis from each participant.

The electroencephalographical (EEG) with a sampling rate of 256 collected throughout the flight were obtained utilizing the Advanced Brain Monitoring (ABM) X-24 system, which included 20 electrodes configured in alignment with the international 10–20 system (Fig. 1 (left)). Electrodes were placed at Fp1, Fp2, F7, F3, Fz, F4, F8, T3, C3, Cz, C4, T4, T5, P3, Pz, P4, T6, O1, Poz, and O2, respectively. Baseline activity and the in-flight data were transferred to a laptop via Bluetooth utilizing the ABM software. Individual start and stop markers were placed at points of interest as noted on the flight pattern or at the discretion of the study team to separate the data for statistical analysis neatly. This information was then referenced with the avionics system’s aircraft flight data.

### Cross-validation of flight maneuver workload

Using a Qualtrics survey, the flight tasks for the scenario were independently rated for their perceived level of workload by (n = 24) FAA-rated aviation faculty members and management pilots from the University of North Dakota to confirm the workload classification of the assigned maneuvers. The purpose of this rating was to cross-validate maneuver workloads from qualified aviation experts for later reference during data analysis. Through this cross-validation process, all flight maneuvers were classified as high, medium, or low workload levels, thus, allowing the study team to determine which time segments of the flight pattern or their respective maneuvers fell into those workload categories.

### EEG pre-processing

EEG-Preprocessing refers to the combination of several methods that convert raw and difficult-to-interpret EEG signals into a suitable and more understandable format for further analysis. Pre-processing is a crucial step for EEG analysis because the recorded signals from the scalp are typically contaminated with noises arising from movement and muscle artifacts, power line noise, and eye movement. Consequently, such noisy EEG signals cannot be a reliable reflection of neural activities. In other words, EEG pre-processing involves eliminating such noises to improve the spatial information of neural activities acquired through the scalp and to prepare it for further analysis.

The proposed pre-processing pipeline consists of four steps, including filtering, an adaptive technique for artifact removal, interpolation, and independent component analysis (ICA) to remove the artifactual components. All the steps are elaborated in the following:

The recorded EEG signals were analyzed using EEGLAB, an open-source MATLAB toolbox^36^ (http://sccn.ucsd.edu/eeglab). A bandpass filter between 1 and 45 Hz was used to remove baseline drift and a portion of noisy signals from EEG. This particular filtering bandwidth maintains the typical frequency bands corresponding to human cognition workload^37^. 1 Hz for the high-pass filtering was used because the leftmost values of the power spectral density (PSD) plot would significantly affect the decomposition outcome^38^. In addition, in terms of signal-to-noise ratio (SNR), high-pass filtering between 1–2 Hz can consistently deliver a strong performance^39^.

**Figure 2:**
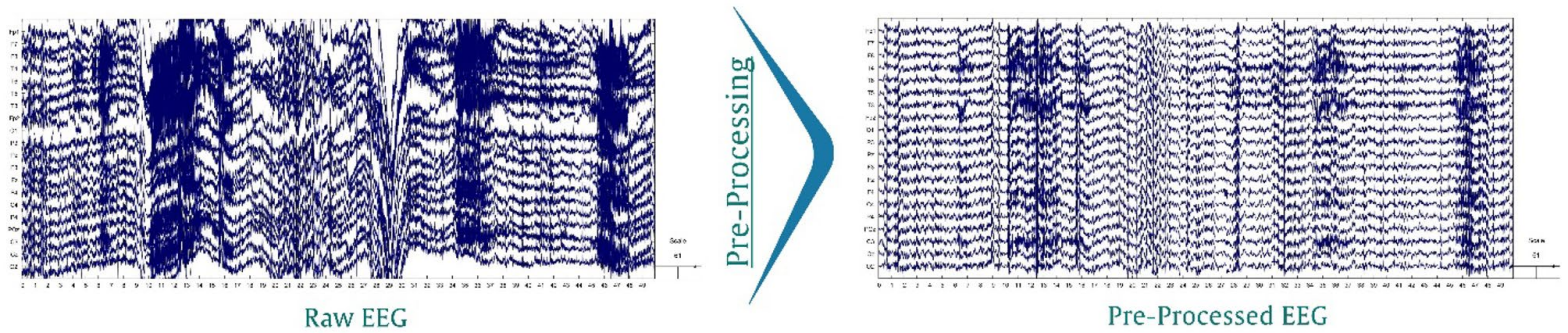
An example of the result of EEG pre-processing steps for 50 s (the x-axis shows time in seconds, and the y-axis shows voltage in units of μV).

For the next step, the filtered data were visually inspected, and noisy channels, dead channels (channel data indicated no activity over longer time periods), muscle activity, and mechanical artifacts in the time domain were removed both manually and using EEGLAB “clean_rawdata” plugin which is an offline version of artifact subspace reconstruction (ASR) technique ^40^. On average, 19.2 (SD = 0.74) EEG channels remained for further analyses (range: 18–20; SD = 0.67). Moreover, to avoid the potential bias toward a hemisphere, all missing channels were interpolated using a spherical algorithm^41^. We skipped the re-referencing to the average step because 20 channel electrodes were used for recording the EEG, and the non-equal distribution of the electrodes could lead to a spatially distorted average. In the next step, Independent Component Analysis (ICA) was computed using the EEGLAB runica function in order to extract independent components (ICs) from scalp electrode signals reflecting maximally statistical independent source time series^42,43^. An equivalent dipole model was calculated for all ICs using a Boundary Element Model (BEM) based on the MNI brain (Montreal Neurological Institute, MNI, Montreal, QC, Canada), which is implemented by DIPFIT routines^44,45^. The ICLabel toolbox^46^ was used as an automatic ICs classification approach to label different brain ICs including the brain, non-brain, eye, muscle, heart, and other sources. Consequently, eye components and the other artifactual components with an assigned probability of higher than 0.8 were selected and eliminated from the data and cleaned EEG signals were used for further processing. The result of pre-processing steps for one subject and a 50-s long EEG is shown in Fig. 2.

### Feature extraction

The feature extraction step was performed using power spectral density (PSD) and log energy entropy. Generally speaking, the former method represents the power distribution into the frequency component of signal^47^, and the latter describes the amount of information carried by a signal or how much randomness is in signal^48^. We considered two minutes (sample size 30,720) of each maneuver for calculating the PSD and log energy entropy. The EEG signals related to each maneuver were transformed into PSD using the Fast Fourier Transform and one-second hamming windows with 50 percent overlap to maintain both temporal and frequency resolution and also to minimize the data loss in the window boundary. Then each EEG channel was divided into four sub-bands based on its frequency range, namely Delta (1–4 Hz), Theta (4–8 Hz), Alpha (8–13 Hz), and Beta (13–30 Hz). Since each EEG sub-band has a different frequency range, the average power spectrum for each sub-band was calculated and used for further analysis. In addition to calculating the PSD for each sub-band, the average power spectral density ratios were also calculated. For instance, the average PSD of the theta band of each electrode in the frontal area is divided by the alpha band of each electrode into parietal and occipital regions (frontal theta/parietal occipital alpha), theta divided by beta for each EEG electrode (theta/beta), and beta divided by alpha + theta for each EEG electrode beta/(alpha + theta).

Similar to the calculation of the PSD, we calculated the log energy entropy for each maneuver and EEG sub-band to extract entropy features using the following equation.

1 $$E= \sum_{n}\mathrm{log}\left({w}_{j,k}^{{n}^{2}}\right),$$

where ${w}_{j,k}^{n}$ are the WP decomposition coefficients.

### Feature selection

The feature extraction step resulted in 228 features from both PSD and log energy entropy of the EEG sub-bands (148 features for PSD and 80 features for log energy entropy). From 148 PSD features, 80 belonged to twenty electrodes and four EEG sub-bands (20 electrodes × 4 EEG sub-bands), 28 features belonged to theta wave of frontal electrodes divided by alpha wave of parietal and occipital electrodes, 20 features belonged to beta wave divided by alpha plus theta for each electrode, and finally 20 features belonged to theta wave divided by beta wave for each electrode. Moreover, the 80 features of log energy entropy belonged to four EEG sub-bands of each electrode.

The number of extracted features in this study is relatively high, and it can not only increase the data complexity and model training time but also by increasing the data variance, increase the chance of model overfitting and negatively impact the performance and accuracy. In other words, the feature selection techniques, by tackling the curse of dimensionality, can improve the learning performance, leading to improving the predictive accuracy and enhancing the comprehensibility of the obtained results. Hence, in this study, after removing the mean from features and scaling them to unit variance (standardization), we employed two feature selection algorithms, namely recursive feature elimination (RFE) and Lasso cross-validation (LassoCV), to choose the most important subset of the original features by removing the irrelevant and redundant features.

In the RFE technique, a classifier can be trained to compute the ranking criterion for all features, and the features with the lowest ranking (weakest features) will be removed^49^. This procedure will be recursively repeated until the number of selected features is reached the predefined desired number. The initial version of the RFE used a support vector machine (SVM) for ranking the features, and in this study, we employed the same setting.

The lasso (Least Absolute Shrinkage and Selection Operator) is the other feature selection technique used in this study, mainly used for regulation and feature selection. In the lasso method, a threshold value (upper bound) is defined for putting a constraint on the sum of the absolute value of the model parameters. To do this, the approach employs a shrinkage (regularization) procedure in which it penalizes the coefficients of the regression variables, thereby shrinking some of them to zero. The variables with a non-zero coefficient following the shrinking procedure are chosen to be part of the model during the feature selection procedure. The purpose of this procedure is to reduce the prediction error as much as possible^50^. In other words, the main goal of the lasso is to minimize the empirical risk subject to ${\left|{\beta }_{L}\right|}_{1}\le {\lambda }_{L}$ for $L=1,\dots .,d$, by finding the β for a given loss function $L$^51^.

2 $$R\left({\beta }_{0},\beta \right)=\sum_{i=0}^{n}L\left({y}_{i},{\beta }_{0}+\sum_{L=1}^{d}{x}_{iL}{\beta }_{L}^{^{\prime}}\right),$$

where x and y are the domain of input and output, respectively, $\beta$ is the regression coefficient, and $\lambda$ is the amount of shrinkage.

More specifically, in this study, we used lasso cross-validation in which the best model can be selected by cross-validation.

### Classification

**Figure 3:**
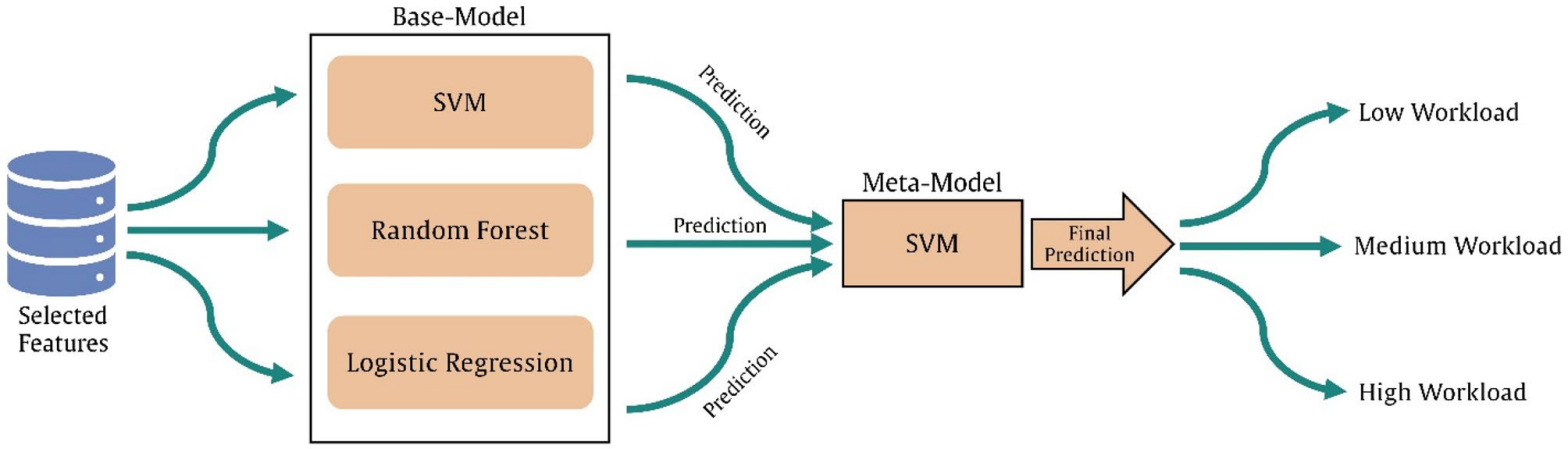
The architecture of the stacking ML model.

Classification refers to a supervised process through which algorithms are intended to learn from one part of data, known as training data, and utilize the learned pattern and information to classify the new unseen part. In this study, the classification task was defined as the determination of different levels of pilots' cognitive workload (low, medium, and high cognitive workloads), which was categorized as a multiclass classification task. We employed a stacking ensemble machine learning algorithm for differentiation between three levels of cognitive workload. Generally, stacking models consist of two levels, including a base-model (level 1) and a meta-model (level 2). Level 1 is composed of different machine learning algorithms (two or more) in which the prediction of each ML model is considered as a new feature and input for level 2. The level-2 which can be similar (or different) to one of the ML algorithms in the base-model tries to learn the best combination of the new features (predictions from level-1) to compute the final prediction. The stacking model provides the opportunity of using the capabilities of a variety of ML models to make a prediction that outperforms any single model. The architecture of stacking ML used in this study is illustrated in Fig. 3.

As can be seen in Fig. 3, Level-1 of our model consisted of three well-known ML classifiers, namely support vector machine (SVM), Random Forest (RF), and Logistic Regression (LR). The output of these algorithms was used as a new feature to feed the SVM in level-2 for the final prediction.

Moreover, k-fold cross-validation (k = 10), in which all the data were split into ten subsets, was used to obtain a more reliable and accurate estimate of the proposed stacking model performance on unseen data and also to prevent the model from overfitting. The k-fold cross-validation iteratively evaluates the model on one of the k subsets (10% in our case) while using the k-1 sunsets for training, and the average of all k-time evaluations will be reported as the final results.

#### Evaluation metrics

To evaluate the performance of the proposed model, including feature extraction, feature selection, and classification algorithms for differentiation between different levels of the pilot's cognitive workload, the confusion matrix of each model was obtained, allowing the calculation of accuracy, precision, recall, and F-score. For each model, the area under the curve (AUC) of receiver operating characteristic (ROC) (AUC-ROC) was also obtained.

In the following, the evaluation metrics are described briefly:

3 $${\text{Accuracy}}\,{ = }\,\frac{{\text{TP + TN}}}{{\text{TP + FP + TN + FN}}}{\text{, Precision}}\,{ = }\,\frac{{\text{TP }}}{{\text{TP + FP }}}{,}\,{\text{Recall}}\,{ = }\,{ }\frac{{{\text{TP}}}}{{\text{TP + FN}}}{\text{, F}}\,{\text{score}}\,{ = }\,{2}\, \times \,\frac{{{\text{precision}} \times {\text{recall}}}}{{{\text{precision}}\,{\text{ + recall}}}},$$

where TP, TN, FP, and FN represent true positive, true negative, false positive, and false negative, respectively. As shown above, accuracy is defined as the number of correct predictions divided by the total number of predictions, precision or positive predictive value is defined as the number of true positives divided by all the positive predictions, and recall, which is also called sensitivity, is defined as the number of true positive predictions divided by the total number of true positives and false-negative predictions. Generally, precision represents the result relevancy, while recall shows the number of truly relevant returned results. Since precision and recall represent different aspects of the classification performance, and in some way, they are complementary, it is rational to combine these two metrics in a single measure^52^. Hence, the F score or F1 score, a harmonic mean of precision and recall, can be a more reliable metric for representing the classifier performance. The AUC-ROC, which is plotted based on true positive rate $\left(\frac{\text{TP}}{\text{TP + FN}}\right)$ (y-axis) versus false-positive rate $\left(\frac{\text{FP}}{\text{FP + TN}}\right)$ (x-axis), considered one of the most important metrics for visualization and monitoring a classifier's performance, shows a classification algorithm's ability to differentiate different classes.

In addition, since we were facing a multiclass classification problem, all the metrics were reported by macro-averaging, in which the corresponding metric for each class was calculated, and then their unweighted mean was reported.

#### Hyperparameter optimization

Hyperparameter optimization or tuning is crucial for designing a high-performance ML-based model and improving its accuracy. In this study, several questions need to be answered for a proper design of the proposed model. For instance, in the feature selection step, the optimum number of the selected features could be, or which estimator could lead to selecting the best subset of the features in RFE. In the classification step, what could be the optimum value for C and gamma in SVM, or the number of trees and criteria in the RF, or more importantly, which combination of ML algorithms could lead to the highest accuracy in the proposed stacking ML model. Conducting trials using different combinations of these vast numbers of hyperparameters could be tedious and cumbersome. Hence, in this study, we employed GridSeachCV, which exhaustively searches over a set of predefined hyperparameter values and evaluates the ML algorithms to achieve the best combination and optimum configuration of the values of the hyperparameters for the given ML model.

## Result

In this paper, the performance of a proposed method, including power spectral analysis and log energy entropy for extracting the meaningful features from four EEG signal sub-bands, two feature selection algorithms (RFE and LassoCV) for selecting the most important extracted features, and stack machine learning algorithm for classification of the selected feature with the aim of determination and classification of different level of pilots' cognitive workload during the flight was evaluated. The results of the proposed method are given and discussed in the following.

It is worth mentioning that the reported results are based on the combination of the tuned hyperparameters using GridSearchCV. In feature selection, for RFE, the SVM with a linear kernel, C = 1 and gamma = 0.1, was selected as the estimator. For LassoCV, the path length was set to 0.001, the number of alphas along the regularization path was determined to 150, the maximum number of iterations was considered 1500, and tolerance for optimization was set to 0.0001. The cross-validation splitting strategy was determined as tenfold cross-validation. Moreover, different numbers of the feature subset were defined (5, 10, 15, 20, 30, 40), and among them, 15 selected features resulted in the highest classification accuracy. For stacking ML model, the GridSearchCV resulted in choosing logistic regression (penalty = l2, tolerance for stopping criteria = 0.0001, and solver = Newton-conjugate gradient), SVM (C = 1, gamma 0.01), and RF (number of estimators = 500, criterion = entropy) as the base model and RF with the same hyperparameters as the meta-model.

### Feature selection results

The top fifteen features out of the 228 total number of features were selected using the RFE and LassoCV, shown in Table 1. As can be seen, PSD of theta, alpha, and beta bands in the right temporal (T4) and parietal, occipital (POz) areas are the important features that were selected using RFE for classification between three cognitive workload levels. More importantly, six features out of the 15 most important features belonged to PSD of frontal theta divided by parietal and parietal occipital area, which can play a crucial role in differentiation between low, medium, and high cognitive workloads. In addition to PSD, six features from the log energy entropy of delta, theta, alpha, and beta bands in both right and left temporal areas (T3, T6, T5) and central (Cz) were selected by RFE as the important feature. In the same way, LassoCv resulted in the selection of 6 features out of 15 for log energy entropy of delta and theta bands in frontal (F7, Fp1), central (C3), and temporal (T6, T5) areas. The other two important features were the PSD of the frontal theta divided by parietal alpha. Further, PSD of the delta, theta, alpha, and beta bands in frontal, temporal, and parietal areas (F8, F4, T3, Pz, and P4) was selected by LassoCV as the other five important features for the classification of three cognitive workload states. Moreover, the ratio of theta and beta bands in temporal and occipital areas (T6, O2) were the other two important features.

**Table 1:** Top fifteen Selected features using RFE and LassoCV.

RFE	LassoCV
Theta band of T4	Log energy entropy of Theta band in C3
Alpha band of POz	Beta band of Pz
Beta band of T4	Theta band of T6/Beta band of T6
Theta band of F7/Alpha band of P3	Delta band of T3
Theta band of F8/Alpha band of POz	Log energy entropy of Beta band in T6
Theta band of Fp2/Alpha band of Pz	Theta band of F8/Alpha band of P3
Theta band of Fp2/Alpha band of POz	Theta band of F4/Alpha band of P3
Theta band of Fz/Alpha band of POz	Log energy entropy of Delta band in T5
Theta band of F4/Alpha band of POz	Alpha band of P4
Log energy entropy of Delta band in T6	Theta band of F8
Log energy entropy of Delta band in T3	Theta band of F4
Log energy entropy of Delta band in C4	Log energy entropy of Delta band in T6
Log energy entropy of Theta band in Cz	Log energy entropy of Delta band in F7
Log energy entropy of Alpha band in T5	Log energy entropy of Delta band in Fp1
Log energy entropy of Beta band in T5	Theta band of O2/Beta band of O2

### Classification results

**Figure 4:**
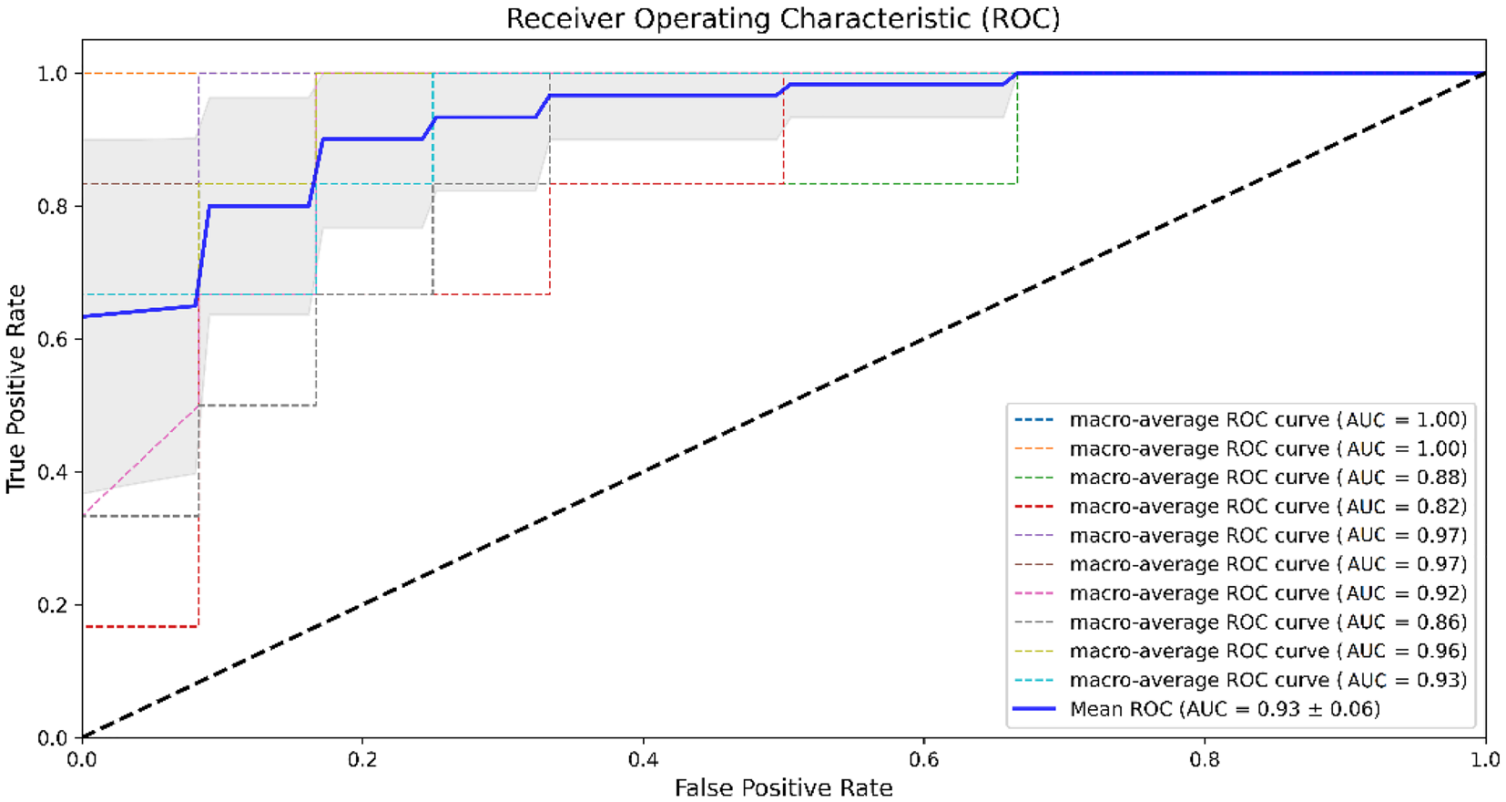
ROC-AUC of the stacking ML model using selected features by RFE.

After selecting the most important 15 features (out of 228), multiclass classification between three pilots' cognitive workload levels was carried out using the stacking ML method to evaluate the model performance. We trained and tested our model using both selected features by RFE and LassoCV with tenfold cross-validation. The highest performance of the model was achieved by feeding the features selected by RFE with an accuracy of 91.67% (± 0.11), precision of 93.89% (± 0.09), recall of 91.67% (± 0.11), and F-score of 91.22% (± 0.12). In addition, the mean ROC-AUC of the model was 0.93 (± 0.06) (illustrated in Fig. 4), which represents the mean ROC-AUC of the macro-average of the ten folds.

**Figure 5:**
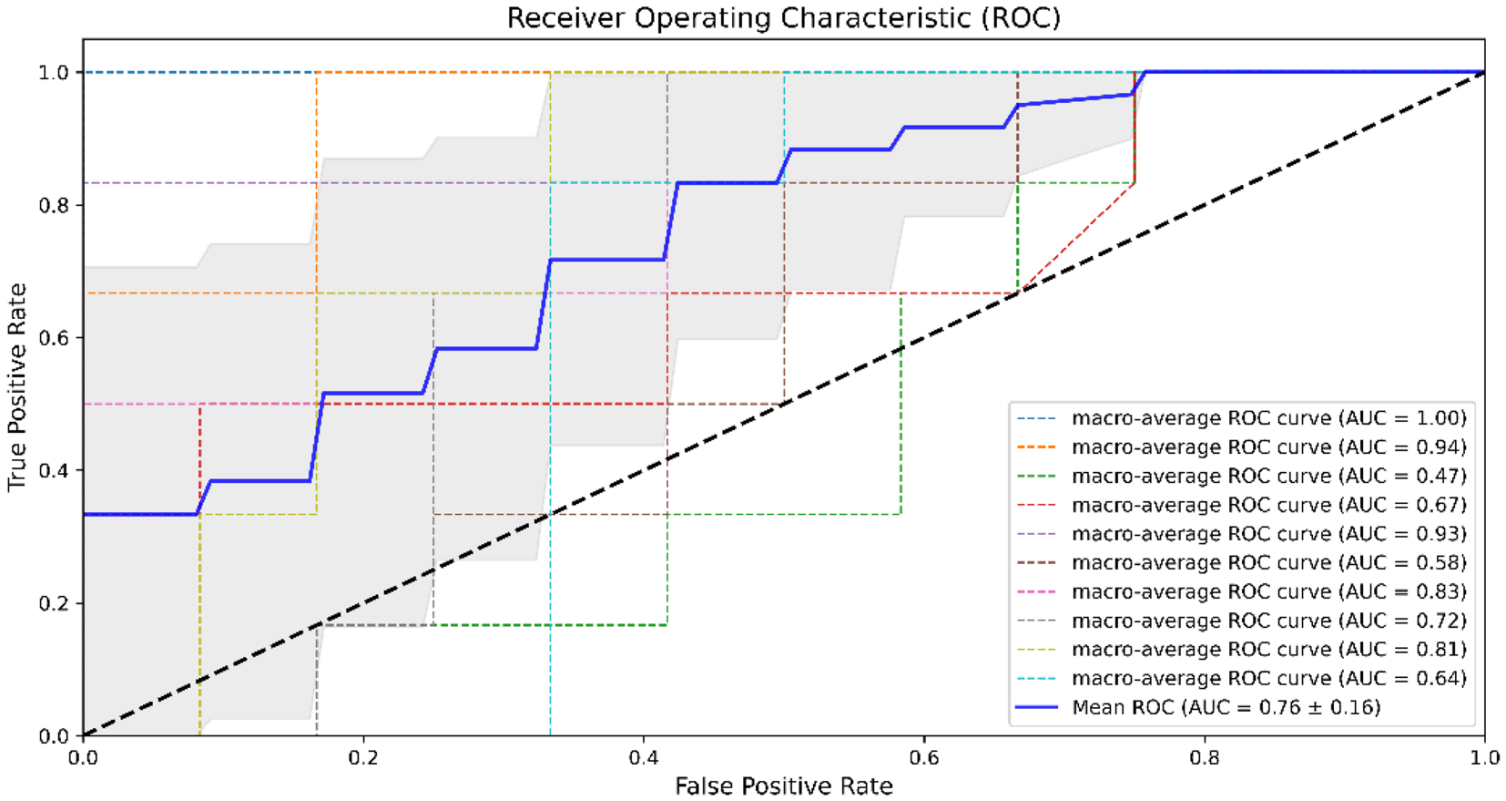
ROC-AUC of the stacking ML model using selected features by LassoCV.

The model showed significantly weaker performance using the selected features by LassoCV than the features selected by RFE. The stacking ML using the 15 features selected by LassoCV resulted in an accuracy of 68.33% (± 0.12), precision of 66.11% (± 0.19), recall of 67.33% (± 0.14), and F-score of 63.89% (± 0.13) for differentiation between the three levels of cognitive workloads. Moreover, the achieved ROC-AUC was 0.76 (± 0.16), which is shown in Fig. 5. There might be two main possible reasons that the LassoCV resulted in a weaker performance. First, choosing the proper regularization parameter is difficult, and improving classification doesn’t necessarily lead to the optimal solution in terms of selecting the best features. Second, when features are correlated, Lasso’s performance degrades since it chooses very few features from each group of correlated features^53^.

## Discussion

Using physiological measures to understand pilot cognition during real or simulated flight operations has been studied using various experimental procedures^9,54–56^. The main aim of the present study was to determine the best method for distinguishing one of three classes of pilot cognitive workload, low, medium, or high, using EEG. A number of factors led to our choice of EEG as a physiological measure of cognitive workload. Some studies suggest that EEG measures are more sensitive measures of different levels of workload than other physiological measures^57–59^. Ocular measures are a problem unless the brightness of the environment is stable and flicker-free^60^. Moreover, the comparison of physiological measures of workload is difficult as tasks have varied across physiological measures^61^. However, a recent study indicates that EEG was more sensitive to differences in workload than measures of gaze entropy^59^. A recent meta-analysis concluded that EEG measures of theta, beta, and alpha are the most sensitive measures of cognitive workload^60^.

The outcome of this analysis was ostensibly to expand the research into flight safety as well as continue the development of the human–machine interface. In the feature extraction step, a wide variety of information was extracted from EEG signals across all brain regions using PSD and log energy entropy. In other words, power distribution, amount of information, and randomness of four EEG sub-bands, including delta, theta, alpha, and beta, from all over the brain scalp were extracted for further investigation. Because of the vast amount of information, two feature selection algorithms, namely RFE and LassoCV, were used to determine the most important features that can lead to the differentiation between pilots' cognitive workload states. To have a more accurate classification between different levels of cognitive workload, a stack machine learning algorithm was employed to not only alleviate the bias of the model but also improve the model performance to improve prediction accuracy^62,63^.

As explained in the result section, the top 15 features selected using the RFE algorithm resulted in higher accuracy, precision, recall, F-score, and mean AUC than the LassoCV. The nine PSD and six log energy entropy were selected as features using RFE, and it is indicated that distributions of power into the EEG sub-bands frequencies combined with the amount of information carried by each sub-band can play a crucial role in distinguishing between different pilots' cognitive workload states. As shown in Table 1, PSD of beta and theta bands in the right temporal (T4) lobe are two of the selected features. The theta band is a well-known indicator of cognitive workload, and an increase in power of theta in three regions of frontal, temporal, and occipital is associated with the level of tasks difficulty^64–66^. More specifically right temporal lobe plays a crucial role in global visual processing^67^. Since the majority of information processed by pilots is obtained by visual scanning of the cockpit displays, visual processing would be the foundation of pilots' decision-making and situation awareness, which is directly related to pilots’ cognitive workload and performance^68,69^.

The beta band in the temporal lobe is considered an indicator of alertness and task engagement and is broadly used for the assessment of cognitive workload^70^. PSD of the beta band shows a significant difference during multi-attribute tasks with different levels of task difficulty^61,71^. So, it can be considered an important indicator for differentiation between pilots’ cognitive workload states due to the fact that the level of complexity of different flight maneuvers such as taxing, takeoff, climbing, approach, or landing is different.

The following important feature was the alpha band's PSD in the parietal occipital (POz) region. Studies showed that there is a correlation between change in alpha power in parietal and occipital regions with the level of complexity and cognitively demanding tasks^23,72,73^. Particularly, by increasing the cognitive workload level, the alpha band's power decreases in the parietal region^74,75^. Hence, this indicator can be used to assess pilots' cognitive workload and indicate mental fatigue and drowsiness during both simulated and actual flights^10^.

Interestingly, in addition to the three discussed features, six features belonged to the PSD of frontal theta (F7, F8, Fp2, Fz, and F4) divided by parietal, occipital alpha. This finding shows this index’s importance for determining the different levels of cognitive workload. Several studies have proved that the index of frontal theta divided by parietal and occipital alpha can be considered an acknowledged indicator of cognitive workload, and it shows a positive correlation with the increasing level of mental workload^14,27^. In other words, an increase in mental workload is assumed to be accompanied by an increase in theta power and a decline in alpha power, which is the basis of this indicator^76^.

Several aviation-related studies investigated the change in power of theta and alpha bands associated with task demands. For instance, in a study conducted by^24^, they monitored subjects' mental workload and fatigue during the operation of a flight simulator, and they found out that the theta band activity increased and the alpha band activity decreased under high workload conditions. In another research by^77^, they employed fifteen pilots who flew a single-engine aircraft on a flight profile with different levels of cognitive workload and showed an association between a high level of cognitive activity and an increase in theta power, and a decrease in alpha power. Altogether, the ratio of frontal theta and parietal occipital alpha seems to be a prominent index for discrimination between different cognitive workload levels. Our proposed method by selecting six features out of fifteen could successfully support the previous findings.

The remaining six features belong to the log energy entropy of all four EEG sub-bands over temporal and central areas (delta in T6, T3, and C4, theta in Cz, alpha and beta in T5). Compared with the other well-known cognitive workload indicators (theta, alpha, and beta, theta/alpha, beta/(alpha + theta), and theta/beta), the delta wave is less investigated for the determination of cognitive workload levels. As stated by^78^, there is a positive correlation between mental task complexity and an increase in delta wave activity, which can be related to the amount of attention devoted to information processing during task performance^70^. Hence, the three selected delta wave indices in this study showed that the information carried by the delta band over temporal and central areas could be valuable features for assessing cognitive workload. The rest of the selected features represent the importance of the information carried by theta, alpha, and beta bands in central (Cz) and temporal (T5) regions which are the other indicators of cognitive workload^70^.

As discussed above, several brain regions and EEG sub-bands are involved in cognitive workload assessment. Moreover, in addition to EEG sub-band power, the amount of information carried by each sub-band also can be a prominent indicator for differentiation between pilots' cognitive workload levels.

## Limitations

Due to data loss and cessation of data collection due to the pandemic, data for this study included EEG and aircraft flight data from 10 participants, each around 1.3 h in total. More participants, including a wider variety of experience levels, would allow for greater generalizability within the aviation pilot community. Additionally, the data collection was in a "live flight" environment. As such, variations in temperature, wind conditions, aircraft performance, and other factors innate to aviation may have subtle, secondary effects on the study outcome. Temperature variations, for example, could affect the perspiration levels of the participant, which could affect the signal quality of the EEG. Variations in wind conditions or turbulence could affect aircraft control and make for easier or more difficult operations from one flight to another. The pattern of the flight scenario was nearly uniform from one data collection event to another; however, clouds, wind conditions, and other traffic may have slightly altered the directions given to the pilot participant in order to ensure normal safety margins and/or compliance with federal aviation regulations were maintained.

## Conclusions and future research

This research aimed to pave the way for a better understanding of variations in pilots’ cognitive workload during actual aircraft flights. PSD and log energy entropy have been employed to extract meaningful information from pilots’ brain activity while performing different maneuvers. Two well-known feature selection algorithms (RFE, LassoCV) and a stacking ensemble machine learning algorithm have been used first to identify the most determining indicators that are able to assess the cognitive workload changes and then classify them into three categories of low, medium, and high workload. Moreover, hyperparameter optimization and tenfold cross-validation were used to improve the model performance and generalization. The achieved results (accuracy of 91.67% (± 0.11), precision of 93.89% (± 0.09), recall of 91.67% (± 0.11), F-score of 91.22% (± 0.12), and the mean ROC-AUC of 0.93 (± 0.06)) proved the validity and efficacy of the proposed approach, for distinguishing different level of pilots' cognitive workload with respect to different flight maneuvers.

Additional research in this domain may inform our understanding of applications of RFE and LassoCV within a subset of electrodes or sensor placements. For example, the future study may include a limited set of EEG electrodes located within an established aviation headset geography (e.g. C3, CZ, and C4) combined with advanced classification techniques to discriminate periods of a low, medium, and high workload. From this information, we may be able to use resultant workload information to observe workload during training operations, observe safety margins during normal or emergency operations or induce system automation changes, such as those described as *augmented cognition.*

Finally, it should be noted that the overarching benefit of this research is the safety of the traveling public. Through a better understanding of pilot cognitive workload during various maneuvers, we can understand how to train pilots, design aircraft, and optimize systems to support successful outcomes. The outcome of this work, similar efforts already completed, and others yet to be undertaken, furnish a broader understanding of the complexities of the human–machine interface. As individuals, governments, and corporations pursue the *benefits* of automation, we must also weigh the role of humans in counterbalancing any authorities we have intentionally or unintentionally delegated to such automation. The ultimate goal of these investigations is to further increase margins within safety–critical environments.

## Supplementary Information

Supplementary Information.

## Data Availability

The datasets used and/or analyzed during the current study are available from the corresponding author upon reasonable request.
