# Supplementary material for: Using machine learning methods and EEG to discriminate aircraft pilot cognitive workload during flight
Source: Sci Rep. 2023 Feb 13;13:2507. doi: 10.1038/s41598-023-29647-0 (PMC9925430; doi:10.1038/s41598-023-29647-0)
Supplement: Supplementary file 1 — Supplementary Information. [file 41598_2023_29647_MOESM1_ESM.pdf]

DATE___________________ SUBJECT ID_______________ Revision 7-15-2019

Task	Maneuver/Procedure	Local Time	Notes
0	Engine Start		
1	Taxi		
2	Takeoff		
3	Foggles on at 1,000' AGL		
4	[reserved]		
5	Turn Eastbound		
6	Climb to 3,500' MSL		
7	Top of Climb (TOC)		
8	Straight and Level (>5min)		
9	Steep Turn LEFT		
10	Steep Turn RIGHT		
11	[reserved]		
12	Initial Vectors towards TVF		
13	Programming TVF ILS31 Approach		
14	Descent to 2,700' MSL		
15	Straight and Level ___ min		
16	Localizer intercept		
17	Glideslope intercept		
18	Minimums		
19	Missed Approach		
20	Turn Westbound		
21	Climb to 4,500' MSL		
22	Top of Climb (TOC)		
23	Straight and Level (>5min)		
24	Steep turn RIGHT		
25	Steep Turn LEFT		
26	[reserved]		
27	Vectors to ILUVE (CKN RNAV 13)		
28	Programming CKN RNAV 13 approach		
29	Descent to 3,000' MSL		
30	Final Approach Course Intercept		
31	Glidepath Intercept		
32	Minimums		
33	Missed Approach		
34	Climb to TPA (1700' MSL)		
35	Landing		
36	Taxi-In		
37	Engine Shutdown (End of Recording)		

NDW07152019
